# Liquid-infused nitric oxide-releasing (LINORel) silicone for decreased fouling, thrombosis, and infection of medical devices

**DOI:** 10.1038/s41598-017-14012-9

**Published:** 2017-10-19

**Authors:** Marcus J. Goudie, Jitendra Pant, Hitesh Handa

**Affiliations:** School of Chemical, Materials and Biomedical Engineering, College of Engineering, University of Georgia, Athens, GA USA

## Abstract

Recent reports on liquid-infused materials have shown promise in creating ultra-low fouling surfaces, but are limited in their ability to prevent bacterial proliferation and prevent platelet activation in blood-contacting applications. In this work, a liquid-infused nitric oxide-releasing (LINORel) material is created by incorporating the nitric oxide (NO) donor *S*-nitroso-acetylpenicillamine (SNAP) and silicone oil in commercial medical grade silicone rubber tubing through a solvent swelling process. This combination provides several key advantages over previous NO-releasing materials, including decreased leaching of NO donor, controlled release of NO, and maintenance of ultra-low fouling property of liquid-infused materials. The LINORel tubing reduces protein adhesion as observed using fluorescence imaging, and platelet adhesion (81.7 ± 2.5%) *in vitro* over a 2 h period. The LINORel combination greatly reduces bacterial adhesion and biofilm formation of two most common pathogens responsible for hospital acquired infections: gram-positive *Staphylococcus aureus* and gram-negative *Pseudomonas aeruginosa* (99.3 ± 1.9% and 88.5 ± 3.3% respectively) over a 7-day period in a CDC bioreactor environment. Overall, the LINORel approach provides a synergistic combination of active and passive non-fouling approaches to increase biocompatibility and reduce infection associated with medical devices.

## Introduction

Blood contacting devices (extracorporeal circuits, catheters, stents, grafts, etc.) are used in thousands of patients every day^1^. Fouling of these devices, either through adsorption of protein leading to thrombus formation, or the adhesion of bacteria resulting in infection, are two of the most common complications seen clinically today^2–4^. The ability to prevent fouling of these devices is critical for the functionality of the device and safety of the patient. While antibiotics and systemic anticoagulation have drastically improved the safety of procedures, researchers continue to strive for a completely biocompatible surface, where passive and active approaches have been developed.

Common approaches to limit the adsorption of proteins (i.e. fibrinogen) include modification of the material surface such as the immobilization of zwitterionic compounds or polyethylene glycol (PEG) and have been demonstrated to provide substantial decreases in fouling of materials both *in vitro* and *in vivo* for bacterial adhesion and thrombus formation^5–8^. Immobilization of heparin have also been shown to decrease thrombus formation; however, none of these strategies have been shown to be 100% effective. A number of limitations remain with these materials, including the leaching of the surface-bound heparin, decreasing the anticoagulation activity over time, and thus require additional systemic heparin to ensure thrombus formation does not occur^9–11^. While it is the current standard in clinical practice, the systemic administration of heparin can cause morbidity and mortality through post-operative bleeding, thrombocytopenia, and hypertriglyceridemia^12^. In the case of extracorporeal circuits, while systemic anticoagulation is required to preserve the patency of the circuit, platelet consumption is still observed and can drop to <40% of the initial value during the first 1-2 h of use^13^. Due to these complications, the systemic administration of anticoagulants is the leading cause of drug-related deaths from adverse clinical events in the United States^14^. Active materials such as antibiotic-releasing or silver-containing catheters are capable of limiting infection, but do not provide any mechanism for reducing thrombus formation^15^. For this reason developing novel materials that possess ultra-low fouling characteristics with materials that can actively kill bacteria and prevent platelet activation and adhesion could provide a drastic advancement in materials for medical devices.

Nitric oxide-releasing (NORel) materials have been developed over the past 30 years after the discovery of NO as an important signaling molecule in a number of biological processes, of which include acting as strong bactericidal and antithrombotic agent^16–18^. To mimic the physiological release of NO from the endothelium, various NO donors (such as *S*-nitrosothiols^18–20^ and diazeniumdiolates^21–23^) have been developed and can be integrated into polymeric materials for localized delivery of NO. Multiple methods have been used to integrate *S*-nitrosothiols such as *S*-nitroso-*N-*acetylpenicillamine (SNAP) into various medical grade polymers, and include physical blending within the polymer^18,24^, immobilization to the polymer backbone^25,26^, or swelling into the polymer matrix^27,28^. However, NORel materials have been shown to have increased protein adhesion^29^, which can ultimately increase the likelihood of bacterial or platelet adhesion on the surface^30^. Despite increases in protein adhesion, NO-releasing materials have been shown to significantly reduce thrombus formation and presence of viable bacteria *in vivo* ^23,24,27,31^. While NO possesses the ability to kill bacteria and prevent platelet activation, decreasing the degree of protein adsorption can act in a synergistic manner to aid in the prevention of thrombosis and bacterial adhesion. Therefore, the development of non-fouling NO-releasing materials can provide further improvements in the overall biocompatibility of existing materials.

Liquid-infused materials take advantage of capillary forces between the infused liquid and the polymer network, creating a low-adhesion interface between the material and the contacting fluid, such as blood. The idea of these slippery liquid-infused porous surfaces (SLIPS) stems from the lining of the gastrointestinal tract, where a mucous layer protects the tissues from colonization by bacteria^32,33^. These materials have shown drastic improvements in the biocompatibility on several common medical polymers, as well as decreasing the adhesion of bacteria to the surface. The efficacy of these SLIPs has been previously demonstrated using tethered perfluorocarbons with a liquid perfluorocarbon held on the surface using capillary forces, as well as the infusion of full medical grade tubing with a biocompatible oil^11,33,34^. It is also important to note that silicone oil has been shown to be nontoxic on the cellular and systemic levels in humans, making it a promising liquid for infusion of SLIPs materials^35^. While these materials provide a passive approach to limit protein or bacterial adhesion, even small amount of adsorbed fibrinogen can lead to platelet activation and adhesion, and ultimately the proliferation of bacteria that can lead to biofilm formation and infection. For example, the presence of thin silicone films has been reported to prevent thrombus formation for short durations^36^, but are not capable of preventing platelet activation and adhesion^11^. The question remains if bacteria or other microorganisms can breach the liquid barrier to the surface, leading to the formation of “beachheads” and enable colonization and biofouling^37^. To overcome bacterial adhesion, the combination of liquid-infused materials and release of a model antimicrobial agent triclosan has demonstrated a synergistic effect of the slippery surface with the active release of antimicrobial agents^37^. One drawback of these materials, however, is these materials do not address issues associated with platelet activation. Incorporating an active release of NO into these materials can aid in the prevention of platelet activation, while also acting as a bactericidal and fungicidal agent to prevent colonization and biofouling on the material surface^38–41^. The use of NO as an antibacterial agent is also attractive as antibiotic-resistant strains of bacteria have been increasingly problematic in the healthcare industry^42,43^.

In this work, fabrication of liquid-infused NO-releasing (LINORel) materials is described, and the synergistic effect of incorporating the NO release with the ultra-low fouling capabilities of liquid-infused materials is demonstrated. The LINORel properties are achieved by using a two-stage swelling process of the NO donor SNAP and silicone oil respectively into medical grade Tygon™ 3350 silicone rubber (SR) tubing. The presence of the infused silicone oil not only provides the desired traits of liquid-infused materials, but also acts in a manner to prevent the burst release kinetics typically associated with NO releasing materials. We demonstrate that these LINORel materials show: (i) reduced adhesion of the blood coagulation protein fibrinogen despite NO release; (ii) reduced platelet adhesion *in vitro*; (iii) and the increased efficacy of preventing biofilm formation of pathogens associated with hospital-acquired infection over a 7 d period. The LINORel approach is the first of its kind to combine the advantages of liquid-infused materials with the active release of an antibacterial, antifungal, and antithrombotic agent, which may aid in future developments of broad-spectrum solution for complications associated with thrombosis and infection associated with medical devices.

## Results and Discussion

### Physical characterization of silicone tubing from SLIPs perspective

Commercial silicone tubing was impregnated with the NO donor SNAP using the previously described swelling method^27^. To demonstrate the incorporation of SNAP has an insignificant effect on the lubricating nature of the silicone oil, sliding angle and oil swelling/deswelling were investigated before and after SNAP incorporation. The infusion of silicone oil into the silicone tubing leads to an expanded state of the polymer tubing, as the polymer chains extend to maximize the polymer-solvent interactions^33^.

**Figure 1:**
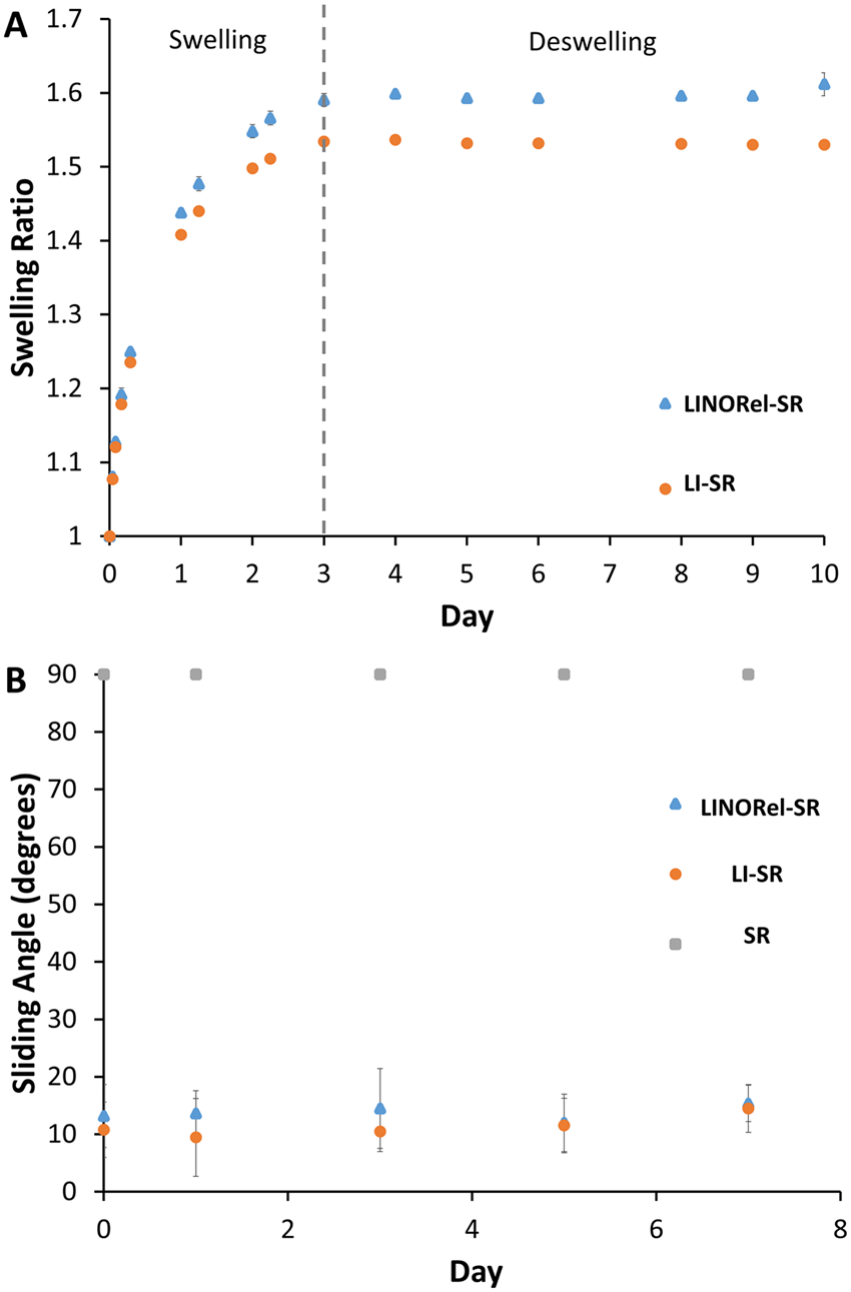
(**A**) Swelling of silicone rubber tubing with silicone oil in control and SR tubing infused with SNAP. Error bars are on the order of data point size and therefore not shown. (**B**) Sliding angle of LI-SR and LINORel-SR tubing over 7 d when stored in phosphate buffered saline at 37 °C.

To observe if the presence of SNAP within the silicone rubber (SR) tubing altered the overall swelling capacity or kinetics, the swelling ratio of oil within the tubing was recorded over 72 hours. The presence of SNAP increased the overall swelling ratio, from 1.53 ± 0.003 to 1.59 ± 0.009 (Fig. 1A, *p* = 0.012). The increase in swelling ratio maybe be attributed to unfavorable interactions between the polymer matrix and crystaline SNAP distrubted throught, leading to higher silicone oil uptake to minimize this interaction. The SR tubing was also capable of maintaining these swelling ratios over the 7 d period at 37 °C. The ability for the tubing to maintain this swelling ratio shows that while the swelling ratio is lower than previously reported^33^, the diffusion of the oil from the polymer matrix has decreased, which coincides with the increased time to reach maximum swelling. However, the decrease in swelling ratio may result in decreased thickness of the liquid layer, impacting the overall performance of the SLIP surface. Therefore, we hypothesize that there may be a compromise in the functionality of the surface with an overall longevity of the infused oil. The full chemical structure of the SR tubing will dictate the overall swelling ratio and could pose the possibility of selecting certain swelling ratio kinetics for the desired application.

All sliding angle measurements were taken using a 2 µL droplet, where the sample was raised slowly at one end and the angle was measured with a digital level. The liquid-infused nitric oxide-releasing (LINORel) SR tubing showed a sliding angle of 13.2 ± 5.5°, compared to oil swollen tubing which had an initial sliding angle of 10.8 ± 2.4° (p > 0.05). Both methods utilizing the incorporation of silicone oil provided drastic decreases in the observed sliding angle when compared to SR controls, which all showed sliding angles > 90°. The sliding angle was observed to slightly increasing over a 7 d period for both NORel-SR and LINORel-SR samples (Fig. 1B). Previous results of infused silicone report sliding angles in the single digits (2.1°)^33^. However, these samples were prepared using the silicone Sylgard 184 and prepared as a flat surface. Therefore, we leave the possibility that the composition of the Tygon™ tubing could lead to increased sliding angle. However, we presume the potential cause of this increase stems from the inability for the commercial tubing to achieve a completely flat surface, creating regions for pinning. It has been shown previously that the SNAP swelling process has minimal effects on the surface morphology of the silicone tubing, and is supported by the similar sliding angles of LI-SR and LINORel-SR tubing^27^. Overall, the drastic decrease between control and the oil infused tubing are substantial in demonstrating the efficacy of the oil to increase the slippery nature of the tubing.

### Characterization of liquid-infused NO releasing silicone rubber from NO perspective

#### Leaching of S-nitroso-acetyl-D-penicillamine

The leaching of NO donors can have detrimental effects on the release characteristics and overall lifetime of the device. This phenomenon is generally associated with a “burst-release” of NO during the initial hours of use. Methods to limit the leaching of physically incorporated NO donors have included the use of hydrophobic polymers^18^. For NO donor-polymer combinations that have minimal leaching (<5% of the total NO donor incorporated), incubation of the device in a solution for an allotted time has been used to control the burst effect when used *in vivo* ^18,31^. While the overall leaching of the donor is low compared to the total loading of the NO donor, the timeframe in which the donor is released to the blood stream can still have systemic effects such as vasodilation and decreases in blood pressure. Therefore, even materials that experience minimal NO donor leaching can still exhibit burst-release characteristics at implantation.

**Figure 2:**
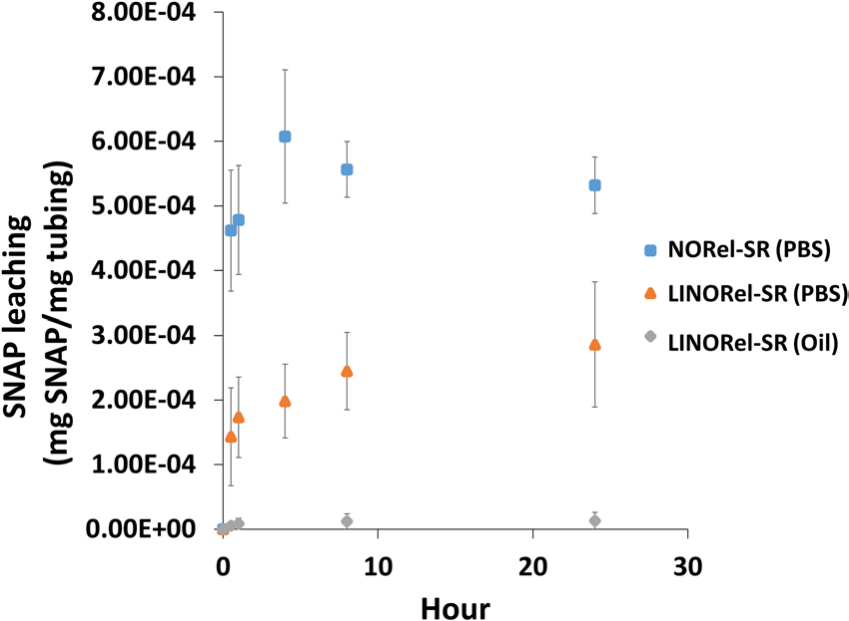
Leaching characteristics of SNAP from NORel-SR and LINORel-SR. Leaching was conducted at room temperature for oil swelling, and physiological conditions (PBS, 37 C). Samples were protected from light at all times.

The leaching of SNAP from the SR tubing was examined using UV-vis spectroscopy during both the oil swelling (72 h) as well as the first 24 h in PBS under physiological conditions (Fig. 2). During the infusion of the tubing with silicone oil, the amount of leached SNAP was observed to not increase after the first 8 h (1.3 ± 0.1 × 10^−5^ mg SNAP mg^−1^ tubing), which may be attributed to the solubility of SNAP (or the base molecule NAP) in the silicone oil. Solubility of SNAP in silicone oil was found to be 0.4 μg mL^−1^. Therefore, increasing the swelling time past 8 h should not have significant effects on the levels of SNAP within the SR tubing when compared to NORel-SR that is stored at room temperature. The stability of SNAP within a polymer matrix has been shown to retain 87% of SNAP activity after 6 months at room temperature^44^. Once placed into the aqueous environment at physiological conditions, the LINORel tubing demonstrated significantly lower leaching levels than of the NORel tubing alone over an initial 24 h period (5.3 ± 0.4 × 10^−4^ mg SNAP mg^−1^ tubing vs. 2.9 ± 1.0 × 10^−4^ mg SNAP mg^−1^ tubing, *p* = 0.02). The total leaching of the LINORel tubing (from both oil and PBS incubation) was reduced by ca. 45% than that of NORel tubing alone.

#### Nitric Oxide release *in vitro*

**Figure 3:**
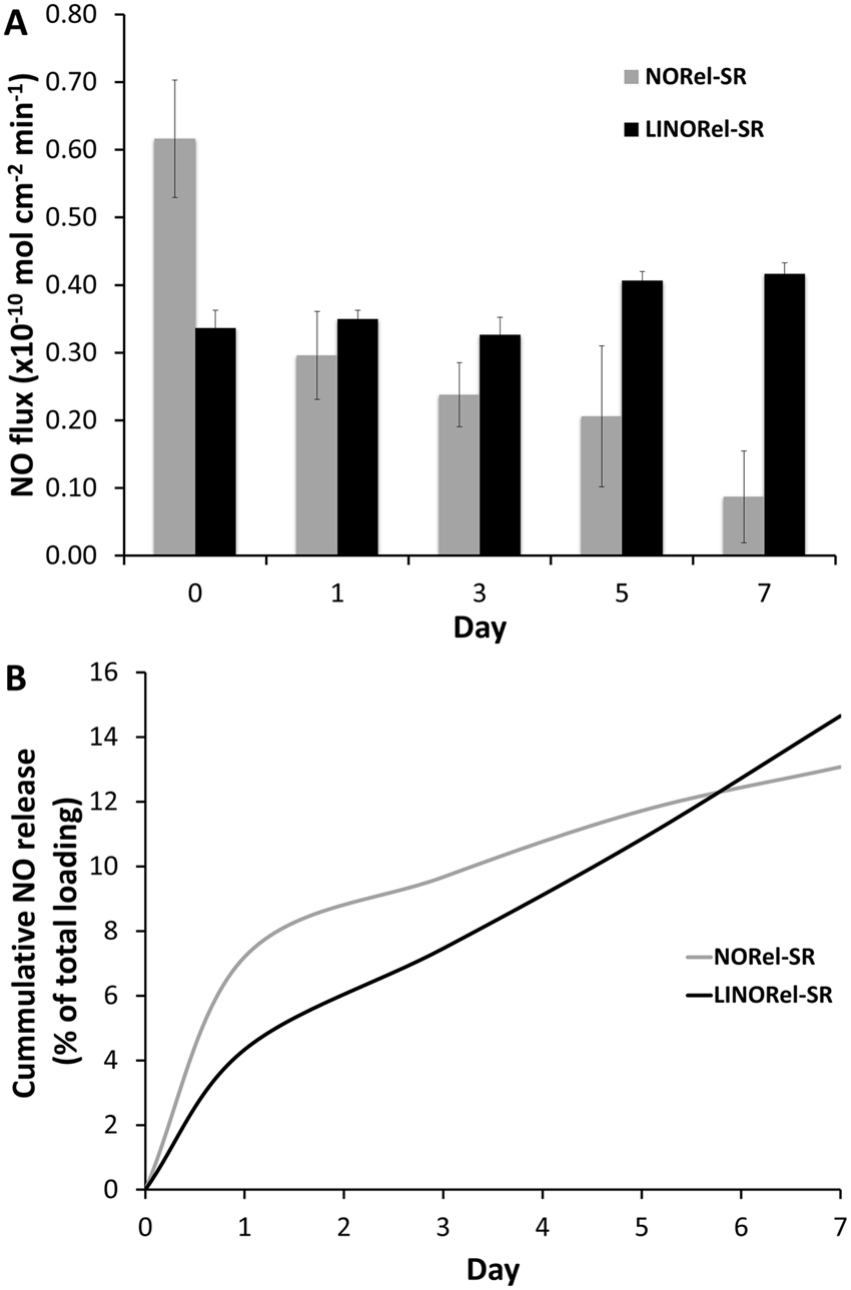
(**A**) Average daily nitric oxide release measures from NORel-SR and LINORel-SR tubing over a 7-day period. Measurements were conducted at 37 °C using a Sievers Chemiluminescence Nitric Oxide Analyzer. (**B**) Cummulative release of NO from NORel-SR and LINORel at physiological conditions due to leaching and degredation of the NO donor.

Nitric oxide release was measured over a 7 d period from both NORel-SR and LINORel-SR using a Sievers chemiluminescence nitric oxide analyzer (Fig. 3A). Release of NO from the SNAP infused tubing exhibited an NO release profile that is consistent with other previously reported materials, showing higher levels of NO initially, and gradually decreasing until reaching a steady state release^18,27,44^. Controlling this burst release is a prime objective for researchers when developing new NO releasing materials, as the burst can be detrimental to the lifetime of the device. The SNAP swelling concentration of 25 mg mL^−1^ was chosen as it was previously shown to provide physiological levels of NO release and significantly increase the hemocompatibility of SR tubing *in vivo* ^27^. However, silicone Foley catheters have been shown to release NO for over 30 d when swollen with 125 mg mL^−1^ SNAP in THF^28^, demonstrating that further optimization of the SNAP swelling concentration can be investigated. The total loading of SNAP using the swelling process was found to be ca. 1 wt% (via chemiluminescence) when a concentration of 25 mg mL^−1^ is used, and is consitent with previous SNAP swelling reports (ca. 5 wt%, 125 mg mL^−1^)^28^. Release rates for the NORel-SR tubing decreased from 0.62 ± 0.09 × 10^−10^ mol min^−1^ cm^−2^ to 0.09 ± 0.07 × 10^−10^ mol min^−1^ cm^−2^ over the 7 d period. The LINORel-SR tubing demonstrated a consistent release over the 7 d period, with initial and final release rates of 0.34 ± 0.03 × 10^−10^ mol min^−1^ cm^−2^ and 0.42 ± 0.06 × 10^−10^ mol min^−1^ cm^−2^, respectively (*p* > 0.05). The while the bactericidal activity was not examine > 7 d, LINORel SR was able to provide this sustained release of NO over a 21 d period, making this a plausible approach for long term applications (Supplementary Figure S1). The cummulative release of NO from the material due to both leaching and degredation of SNAP to NAP is shown in Fig. 3B as a percentage of the total SNAP loaded. Therefore, the incorporation of the silicone oil not only assists with non-fouling capabilities of the tubing but provides a more controlled NO release from the donor as well. This can be attributed to the silicone oil preventing the hydration on the silicone tubing, which can lead to faster release of NO from the donor^16,18^. Further optimization is needed on the swelling method can be done on the concentration of SNAP within the swelling solution as the release will not only be governed by the total amount of SNAP loading, but also the crystal structure within the silicone matrix^45^. It is also possible that the crystal structure of SNAP within the polymer may be tunable using the rate of solvent evaporation, and may also be influenced by the degree of crosslinking within the polymer. Possiblities for improving the activity of the LINORel SR by increasing the NO release could involve integration of metalic complexes such as copper into the silicone matrix^46^, or for non-implanted applications such as extracorporeal circuits stimulation with light may be applied to modulate the NO release^25,47^.

### Assessment of fibrinogen adsorption *in vitro*

One common method for assessing the hemocompatibility of materials *in vitro* is to examine the ability of the material to resist protein adhesion, more specifically, fibrinogen. The conversion of fibrinogen to fibrin in the common pathway of the coagulation cascade, and the adhesion of platelets through GpIIb/IIIa, lead to the formation of thrombus on the material surface. While the orientation of fibrinogen adsorption has been shown to determine the degree of platelet adhesion, limiting protein adhesion regardless of orientation is generally considered to be an improvement in the hemocompatibility of a material^48^. Apart from aiding in the formation of thrombus, surface bound protein has been shown to increase the level of bacterial adhesion, increasing the chance of biofilm formation and infection^30^. Although NO releasing materials have been shown to significantly reduce platelet activation and adhesion, they have also been shown to adsorb higher levels of fibrinogen^29^. Therefore, developing non-fouling NO-releasing materials could provide drastic improvements in the overall hemocompatibility and antibacterial nature of these materials.

**Figure 4:**
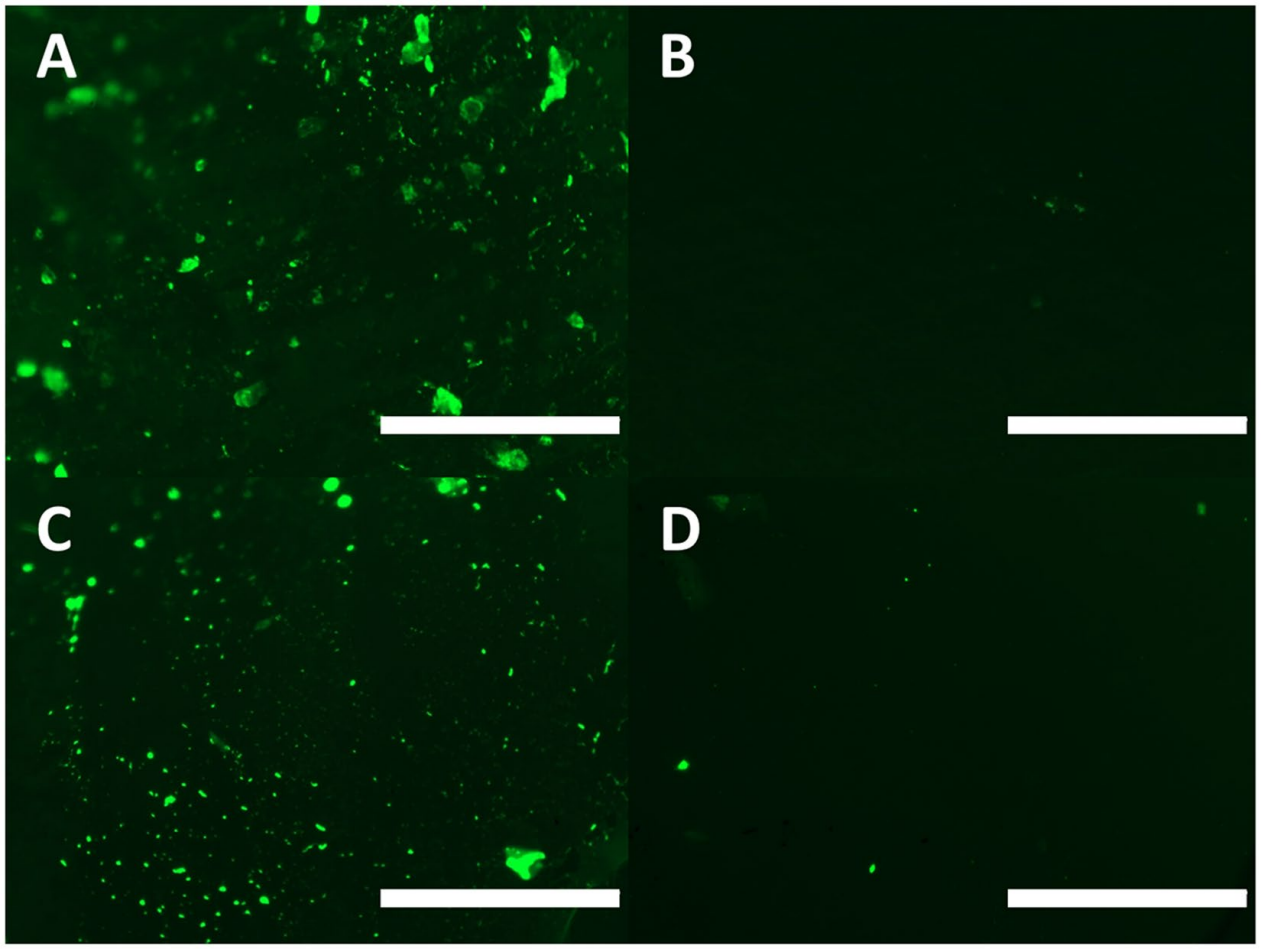
Assessment of protein adhesion (FITC labeled fibrinogen) after 2 h incubation on (**A**) SR (**B**) LI-SR, (**C**) NOrel-SR, (**D**) LINORel-SR. Scale bar represents 250 µm.

To examine if the infusion of silicone oil to provide a slippery surface could overcome the increased protein adhesion observed on NO-releasing materials, 2 h exposure to FITC-labeled fibrinogen was conducted at 37 °C (Fig. 4). NORel-SR was observed to adsorb comparable amounts of fibrinogen compared to the control SR tubing, which coincides with previously reported results^29^. The presence of the infused oil was observed to greatly reduce protein adhesion in both LI-SR and LINORel tubing. Therefore, the infusion of silicone oil was successful in drastically reducing the adsorption of fibrinogen despite NO release. The adhesion of the protein of the surface was not measured quantitatively, although MacCullum *et al*. has reported that the measured bacterial adhesion on silicone oil infused tubing can vary drastically with the method that the material is washed^33^. In this study, the authors show that with no wash, ca. 90% reductions in biofilm formation was observed with LI-SR alone; however, it reduced to nearly 100% with both 5 s and 5 min wash times under high shear. The infinite dilution of this method for washing of the material surface ensures minimal shear on the material surface, and therefore we believe represents the highest levels of protein adsorption that would be seen, with much of the protein loosely bound to the surface. The efficacy of these materials in long-term exposure to protein-rich environments is currently under investigation by our group and will vary highly with duration of exposure and applied shear forces, ultimately governing the thickness of the thin film protecting the surface.

### Assessment of porcine platelet adhesion *in vitro*

**Figure 5:**
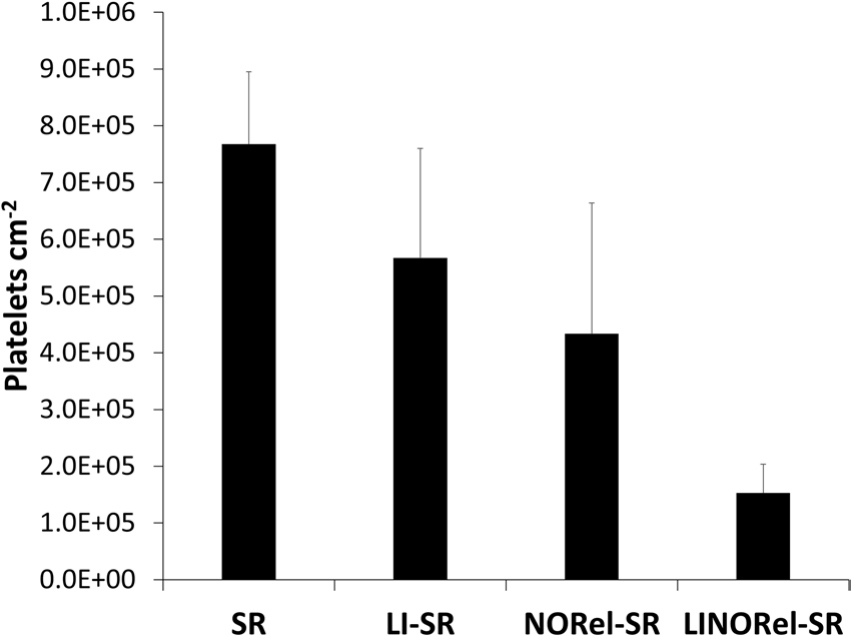
Degree of platelet adhesion on various silicone tubing after 2 h exposure to porcine platelet rich plasma as measured using an LDH quantification assay.

Sections of the various modified Tygon™ tubing were exposed to fresh porcine PRP for 2 h at 37 °C under mild rocking, where the number of adhered platelets were quantified using a Roche LDH assay (Fig. 5). The presence of the infused oil provides a lubricating layer, separating the material surface from the liquid to be in contact, as well as drastically reducing the surface roughness^11,33,34^. Presence of the SLIP surface resulted in 27% reduction in the overall adhered platelets (7.76 ± 1.70 × 10^5^ platelets cm^−2^ vs 5.67 ± 2.58 10^5^ platelets cm^−2^, *p* > 0.05), while NORel surfaces saw reductions near 44%. Similar reductions in platelet adhesion have been reported for other liquid infused surfaces when exposed to whole blood for 30 min, containing 0.25 U mL^−1^ heparin^11^. The increased platelet adhesion observed in this study can be attributed to the increased exposure time and absence of anticoagulant. The combination of infused oil with a NO releasing donor molecule further reduced the degree of platelets adhered to 81.7 ± 2.5% of control silicone rubber tubing (7.76 ± 1.70 × 10^5^ platelets cm^−2^ vs 1.52 ± 0.68 × 10^5^ platelets cm^−2^, *p* = 0.03). The LINORel combination was able to significantly reduce platelet adhesion when compared to LI-SR alone (73.1%, *p* = 0.03), and may be attributed to the presence of the silicone oil as it does not prevent platelet activation. However, the LINORel combination did not provide a significant decrease in platelet adhesion when compared to NORel-SR alone (*p* > 0.05). Washing of each material was done through infinite dilution of the well plate, and therefore provided minimal shear at the material interface. The effectiveness of the washing of these liquid infused materials is highly depended on the shear rate and time of wash, and can attribute to higher platelet counts observed^33^. Few dual-action materials incorporating NO release have been developed, with even fewer examined for platelet adhesion *in vitro*. Kipper *et al*., developed a glycoclyx-inspired NO releasing material on titanium to mimic the natural endothelium, where NO release was provided by nitrosated chitosan thioglycolic acid^49^. These materials showed similar reductions in platelet adhesion to the LINORel tubing when examined using scanning electron microscopy after 2 h exposure to human blood plasma containing platelets and leukocytes. However, the NO release from the materials decreased to below 0.01 × 10^−10^ mol min^−1^ cm^−2^ within 20 minutes. The combination of NO with zwitterionic polycarboxybetaine coatings have been reported by Cook *et al*. with similar platelet adhesion to the LINORel materials describe with 93.1 ± 1.3% reductions in platelet adhesion using NO delivery through a permeable polydimethylsiloxane membrane. While this combination is highly effective, the requirement for sweep gas may limit the direct application. Therefore, the combination of the extended non-fouling nature from the SLIP surface with controlling of the NO release profile make the LINORel approach a promising for long term blood-contacting applications.

### *In vitro* 7 d bacterial adhesion and viability analysis in a continuous flow CDC bioreactor

Development of novel materials to reduce bacterial adhesion and growth on materials are generally achieved using one of or a combination of two parameters: (i) the surface characteristics of the material (chemical and physical), and (ii) the antibacterial efficiency attributed to it via the antimicrobial agent. The LINORel approach looks to address each of these parameters by combing an active release of and antibacterial agent (NO) with a liquid-infused surface. While the infused silicone oil provides a super slippery hydrophobic surface for preventing attachment of bacteria on the polymer surface in an environment with shear force (such as CDC bioreactor), the free radical NO provides bactericidal action via lipid oxidation, denaturation of enzymes,and deamination of DNA^50^. To examine the long-term efficacy of these materials to prevent biofilm formation, exposure to two common pathogens associated with healthcare-acquired infections was done in a CDC bioreactor over a 7 d period with gram-negative *P*. *aeruginosa* and gram positive *S*.*aureus*, where *P*. *aeruginosa* causes 10–15% of nosocomial infections worldwide^33,51^.

**Figure 6:**
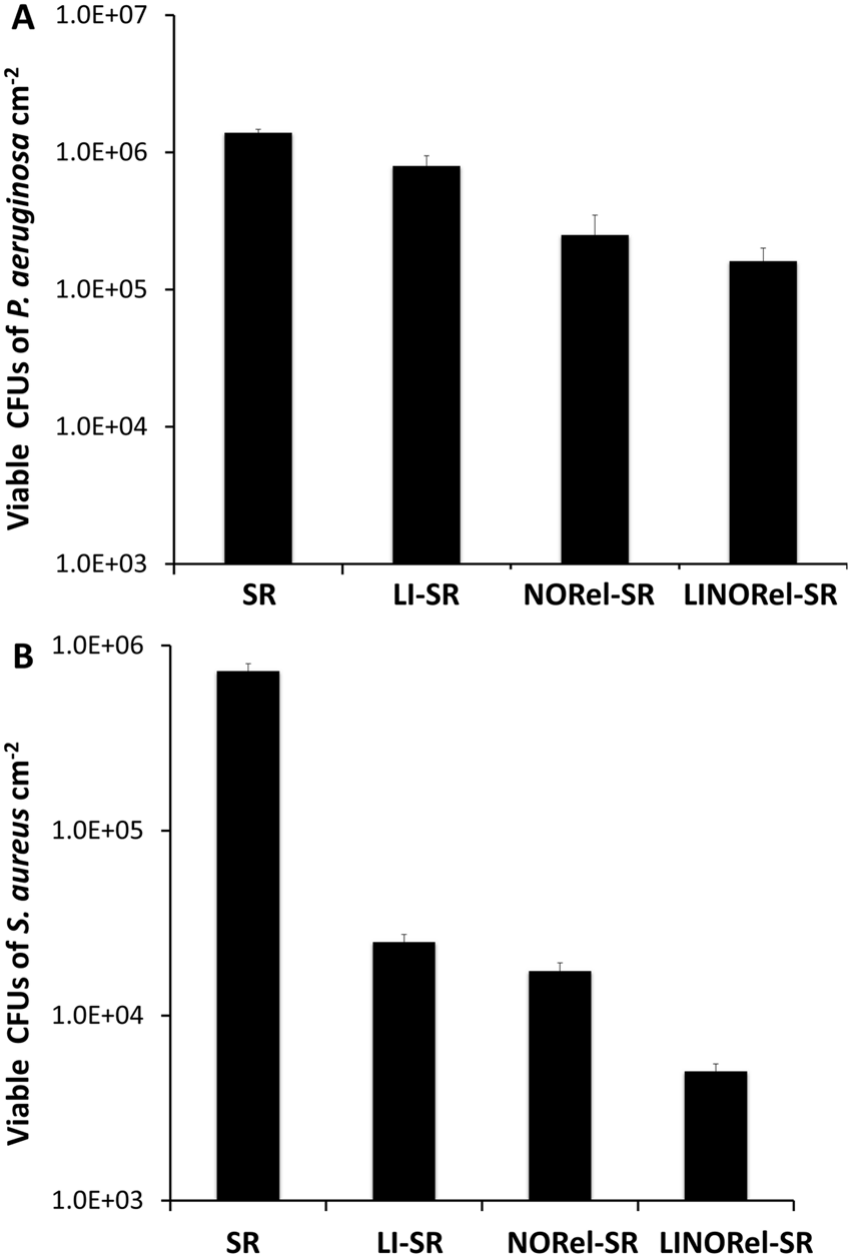
Viable bacteria on various silicone tubing after 7 d bacteria exposure in a CDC bioreactor.

In a similar fashion to the protein adhesion studies mentioned above, minimal washing of the surface was done as to not detach loosely bound bacteria from the material surface through shear forces prior to the intentional detachment via homogenization of the film. Figure 6 graphically represents the CFU cm^−2^ attached on the surface of each of modified tubing. Efficacy of each tubing modification is summarized in Table 1.

**Table 1:** Efficacy of each modification of SR on reducing bacterial adhesion and viability over 7 d in CDC bioreactor.

	*S. aureus*				*P. aeruginosa*			
	SR	LI-SR	NORel-SR	LINORel-SR	SR	LI-SR	NORel-SR	LINORel-SR
CFU × 10^5^ cm^−2^	7.29 ± 1.11	0.25 ± 0.02	0.17 ± 0.04	0.05 ± 0.01	13.92 ± 1.22	7.95 ± 0.53	2.49 ± 0.02	1.61 ± 0.14
Reduction vs. SR (%)	—	96.57 ± 0.3	97.61 ± 0.62	99.31 ± 0.15	—	42.89 ± 3.80	82.08 ± 0.18	88.44 ± 1.01
*p* value vs. SR	—	0.007	0.008	0.007	—	0.004	0.002	0.003

The presence of the infused silicone oil alone reduced CFU cm^−2^ of *S*. *aureus* by 96.5 ± 0.30% and *P*. *aeruginosa* by 42.8 ± 3.8% after 7 d in the CDC environment. The drastic difference in the ability for LI-SR to prevent the attachment of *P*. *aeruginosa* can be attributed to the differences in the structure of the bacteria, and demonstrate the need for an active release of a bactericidal agent such as NO when used in long-term applications. Previously, infusion of silicone oil was shown to reduce *P*. *aeruginosa* adhesion to medical grade silicone by >90% after 48 h exposure^33^. Many of the interactions of bacteria with liquid infused materials are not fully understood. This increase in *P*. *aeruginosa* adhesion at day 7 could stem from the proliferation of few bacteria that had adhered, or overcoming the liquid layer with extended exposure. NORel-SR achieved 97.6 ± 0.6% and 82.1 ± 0.2% reductions against *S*. *aureus* and *P*. *aeruginosa* respectively. The NO flux of the tubing dropped from 0.62 ± 0.09 × 10^−10^ mol min^−1^ cm^−2^ to 0.09 ± 0.07 × 10^−10^ mol min^−1^ cm^−2^ from the initial release to day 7, therefore it would not be unreasonable to predict that the bacterial killing was much higher initially, decreasing over time due to gradual decrease in NO flux. The bacterial killing ability of the NO releasing tubing matches with the previous reports where the bactericidal activity of NO has been demonstrated against *S*. *aureus*, *E*. *coli*, *Candida albicans*, *L*. *monocytogenes*, *E*. *faecalis* and *A*. *baumanni* ^22,41,52,53^. However, both the ability of the liquid infused surface to reduce bacterial adhesion and NO to provide bactericidal activity can vary between bacterial strains, and should be investigated further with the bacterial strain that will apply to the intended device. The NO based strategy to prevent infection is not expected to stimulate resistance in the bacterial strains due to its rapid mode of action and very short half-life (<5 sec) unlike antibiotics and silver nanoparticles^22,54^. Further developing these materials to release levels of NO at the upper end of physiological limits would be expected to provide further reductions in long-term viable bacterial adhesion. LINORel-SR achieved a 99.3 ± 1.9% reduction in gram-positive *S*. *aureus* and 88.5 ± 3.3% reduction in *P*. *aeruginosa* population on LINORel tubing (CFU mL^−1^) as compared to control SR tubing, and was significantly more effecitvive than LI-SR or NORel-SR tubing alone (Table 2). The effect of the LINORel combination is clearly demonstrated, where reductions of the combination are near the reductions observed when comparing a singular modification to the unmodified SR.

**Table 2:** Comparison of LINORel-SR tubing to LI-SR and NORel-SR after 7 d in CDC bioreactor.

	*S. aureus*		*P. aeruginosa*	
	LI-SR vs. LINORel**-SR**	NORel-SR vs. LINORel**-SR**	LI-SR vs. LINORel**-SR**	NORel-SR vs. LINORel**-SR**
Reduction vs. LINORel-SR (%)	80.0 ± 4.7	71.3 ± 4.9	73.2 ± 2.2	35.5 ± 5.7
*p* value vs LINORel-SR	0.001	0.044	0.003	0.004

Integration of biocides into materials to provide antibacterial activity to SLIPs surfaces have been reported by using the combination of triclosan with infused silicone oil in polyethyleneimine/poly(2-vinyl-4,4-dimethylazlactone) (PEI/PVDMA) multilayers, and showed ca. 80% reduction in *C*. *albicans* after three sequential 24 h exposures to 1 mL cell suspension (10^6^ CFU/mL)^37^. However, the biomimetic nature of NO releasing materials is attractive with the emergence of antibiotic-resistant strains of bacteria, as well having antithrombotic properties. Dual action mechanisms to increase the bactericidal activity of NO releasing materials using the combination of NO release with metallic ions^46,55^, quarterinary ammonium compounds^56,57^, antibiotics and antimicrobial peptides^58^ have also been investigated. While the combination of bactericidal agents may provide higher bactericidal activity in the short-term, these materials can experience decreases in efficacy with fouling of the material surface. Therefore, adding bactericidal activity to SLIP materials can be advantageous in protein-rich or bacterial-rich environments where the surface can be comprimised quickly.

### Cytocompatibility of LINORel-SR tubing

The CCK-8 assay was performed to the demonstrate the absence of any toxic response of the leachates from NORel-SR and LI-SR tubing towards mouse fibroblast cells. The results demonstrated that neither NORel-SR or LI-SR, nor their combination in the used dosages,, is cytotoxic to the mammalian fibroblast cells. In the past SNAP and Si-oil has been used individually as an active strategy to control the growth of bacteria on the polymeric surface, however, this would be the first report to show that the leachates from the applied concentration of SNAP and Si-Oil intergrated in the tubing is not cytotoxic to the mammalian cells but still very effective in terms of bacterial inhibition and preventing platelet adhesion

**Figure 7:**
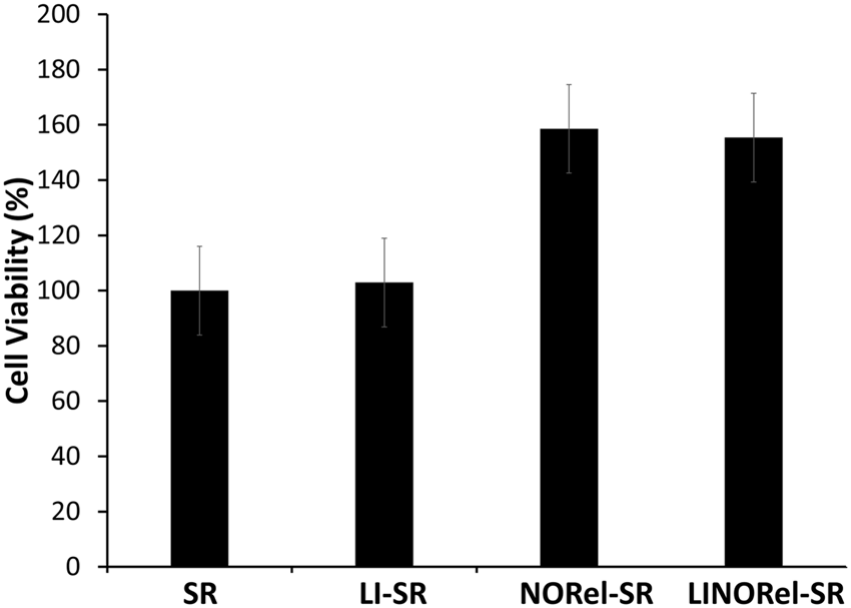
Cytocompatbility and cell growth support of various infused SR tubing towards mouse fibroblast cells in 24 h study.

The results demonstrated that not only the leachates not caused any cytotxic respeonse but at the same time also promoted the proliferation of the mouse fibroblast cells. This can be manly due to the cell proliferationg potential of NO that would have released as a result of putting the tubes in contact with cell culture media. Figure 7 shows the cell proliferation capacity of NO-releasing silicone tubes. It was previously shown that SNAP incorporation in the medical grade polymer resulted in no cytotoxicity in a 24 h study *in vitro* ^44,46^. This is in line with the recent studies which demonstrated endogenous NO has to be important in mammalian cell proliferation. Ziche *et al*., reported that NO induces endogenous basic fibroblast growth factor (bFGF) resulting in upregulation of urokinase-type plasminogen activator (uPA) in coronary venular endothelial cells (CVECs) ultimately resulting in the proliferation of endothelial cells^59^. Another study has shown similar results where endogenous NO was shown to cause an increase in proliferation of endothelial cells from postcapillary venules by promoting DNA synthesis in these cells^60^. However, the current study demonstrated the cell proliferation via NO release from the leachate solution as a result of soaking tubing samples in DMEM medium for 24 h. Theoretically, the NO flux should have been released in the physiological range to show the proliferative response as shown in this study. A further studyis needed to measure the NO flux in the cell culture medium as a result of leachingwhich significantly increased the fibroblast cell proliferation by 60% as compared to control cells without external NO supply (p < 0.05).

## Conclusion

In this work, the combination of liquid-infused slippery surfaces was combined with NO-releasing capabilities in commercial medical grade silicone rubber tubing through the infusion of silicone oil and the NO donor SNAP. The presence of SNAP in the silicone matrix had no significant negative effects on the slippery nature of the surface, with no significant changes in the swelling ratio or sliding angle over 7 d. However, the infusion of silicone oil assisted in the controlled release of NO due to limiting the hydration of the SR. Silicone tubing infused with SNAP showed a decrease in NO-release from 0.62 ± 0.09 × 10^−10^ mol min^−1^ cm^−2^ to 0.09 ± 0.07 × 10^−10^ mol min^−1^ cm^−2^ over the 7 d period, while the LINOrel-SR tubing showed a much more constant release of 0.35 ± 0.03 × 10^−10^ mol min^−1^ cm^−2^ and 0.42 ± 0.06 × 10^−10^ mol min^−1^ cm^−2^. The infusion of silicone oil reduced fibrinogen adsorption over a 2 h period for both LI-SR and LINORel-SR tubing. Bacterial adhesion was investigated over a 7 d period using a CDC bioreactor, where 99.3 ± 1.9% and 88.3 ± 3.3% reductions in viable cell adhesion were observed for *S*. *aureus* and *P*. *aureginosa*, respectively. The combination of SNAP and silicone oil was confirmed to be non-cytotoxic towards mammalian fibroblast cells, while resulting  in the proliferation of mammalian cells due to the possible presence of NO in the leachouts. Overall, the results suggested that the infusion of SNAP and silicone oil into commercial silicone tubing can potentially increase the biocompatibility for medical applications while preventing infection.

## Experimental Section

All methods were performed in accordance to the University Committee on the Use and Care of Animals, and with university and federal regulations.

### Materials

*N*-Acetyl-*D*-penicillamine (NAP), sodium chloride, copper chloride, L-cysteine, potassium chloride, sodium phosphate dibasic, potassium phosphate monobasic, ethylenediaminetetraacetic acid (EDTA), tetrahydrofuran (THF), and sulfuric acid were purchased from Sigma-Aldrich (St. Louis, MO). Methanol, hydrochloric acid, silicone oil, and sulfuric acid were obtained from Fisher Scientific (Pittsburgh, PA). Saint-Gobain™ Tygon^TM^ Formula 3350 silicone rubber (SR) tubing was purchased from Fisher Scientific (Pittsburgh, PA). All aqueous solutions were prepared with 18.2 MΩ deionized water using a Milli-Q filter (Millipore Corp., Billerica, MA). Phosphate buffered saline (PBS), pH 7.4, containing 138 mM NaCl, 2.7 mM KCl, 10 mM sodium phosphate, 100 µM EDTA was used for all *in vitro* experiments. Trypsin -EDTA and Dulbecco’s modification of Eagle’s medium (DMEM) were obtained from Corning (Manassas, VA 20109). The antibiotic Penicillin-Streptomycin (Pen-Strep) and fetal bovine serum (FBS) were purchased from Gibco-Life Technologies (Grand Island NY 14072). The Cell Counting Kit -8 (CCK-8) was obtained from Sigma-Aldrich (St Louis MO 63103). The bacterial strains of *Pseudomonas aeruginosa* (ATCC 27853), *Staphylococcus aureus* (ATCC 6538) and 3T3 mouse fibroblast cell line (ATCC 1658)were originally obtained from American Type Culture Collection (ATCC).

### SNAP Synthesis Protocol

SNAP was synthesized using a modified version of a previously reported method^61^. Briefly, an equimolar ratio of NAP and sodium nitrite was dissolved in a 1:1 mixture of water and methanol containing 2 M HCl and 2 M H_2_SO_4_. After stirring, the reaction vessel was cooled in an ice bath to precipitate the green SNAP crystals. The crystals were collected by filtration, rinsed with water, and dried under ambient conditions. The reaction mixture and resulting crystals were protected from light at all times.

### Preparation of NORel and LINORel tubing

The SNAP swelling solution was prepared by dissolving SNAP in THF using a concentration of 25 mg mL^−1^ as found previously to provide an optimized NO-release^27^. The Saint-Gobain^TM^ Tygon^TM^ SR tubing was soaked in the SNAP swelling solution for 24 h. The tubing was removed, briefly rinsed with PBS, and dried for 48 h under ambient conditions to allow the excess THF to evaporate. After drying, the tubing samples were placed in a 20 mL vial with DI H_2_O, and placed in a Fisher Scientific 1.9 L sonicating bath for 5 min to remove any crystalized SNAP from the surface of the tubing. The tubing and swelling solutions were protected from light throughout the swelling process. Infusion of silicone oil for LINORel tubing was then acheived through incbuation of NORel SR tubing in silicone oil for 72 h at room temperature, and protected from light.

### Nitric Oxide Release and Total SNAP Loading

Nitric oxide release from the silicone tubing was measured using a Sievers chemiluminescence Nitric Oxide Analyzer (NOA), model 280i (Boulder, CO). A section of the NORel-SR or LINORel-SR tubing (1 cm) was placed in 4 mL PBS with EDTA buffer at 37 °C. Nitric oxide purged from the submerging buffer through bubbled nitrogen and was continuously swept from the headspace of the sample cell with a nitrogen sweep gas to the chemiluminescence detection chamber. The nitrogen flow rate was set to 200 mL/min with a chamber pressure of 6 Torr and an oxygen pressure of 6.0 psi. The NO-release from samples is normalized by the surface area using the flux unit (×10^−10^ mol cm^−2^ min^−1^). Both NORel-SR and LINORel -SR samples were incubated at 37 °C in 4 mL PBS with EDTA between NO release measurements to maintain physiological conditions. The buffer was changed daily as to ensure the buffer was not saturated with either SNAP nor silicone oil.

Total loading of SNAP using the swelling process was measured by incubating a small section of the NORel-SR tubing (10–20 mg) in a solution of 50 mM CuCl_2_ and 10 mM L-cysteine at 37 °C^28^. The addition of L-cysteine aids in the catalysis of Cu^2+^ to Cu^+^, which is responsible for the catalytic release of NO from RSNOs such as SNAP^62^. Release rates of NO were then integrated over the duration of the measurement to determine the total NO released.

### Oil swelling

Swelling and deswelling characteristics of the silicone tubing were investigated. For swelling, silicone tubing and NORel-SR were submerged in silicone oil (Alfa Asar). The mass swelling ratio can be defined as the ratio of the mass of the infused polymer (*M*_*i*_) and the mass of the polymer initially (*M*_*0*_) (equation 1).

1 $${Swelling}\,{ratio}=\,\frac{{{\boldsymbol{M}}}_{{\boldsymbol{i}}}}{{{\boldsymbol{M}}}_{0}}$$

Deswelling of the oil from the respective tubing was examined through incubation of the swollen tubing in PBS with EDTA at 37 °C under mild agitation on a Medicus rocker.

### Sliding angle characterization

A sliding stage with a digital protractor was used to measure the sliding angle of a 2 µL droplet of water on the surface of each silicone substrate. Tubing samples (ca. 5 cm in length) were cut longitudinally and mounted onto a glass slide to create a flat sheet of silicone. Samples were gently washed with DI H_2_0 and air-dried with nitrogen to remove any dust or contaminants that were initially on the surface. For each measurement, the angle of the sample was slowly increased until the droplet was observed to slide along the surface, and the angle was recorded using a digital protractor. Each surface was measured at 6 different randomly selected areas. Samples were stored in 50 mL conical tubes containing 40 mL of PBS with EDTA, and maintained at 37 °C in a Thermo Fisher water jacketed incubator, under mild agitation on a Medicus blood rocker. The buffer was replaced after each measurement to avoid saturation of the oil in the incubating buffer. Samples were gently blown dry with nitrogen after being removed from the incubating buffer to ensure any water on the surface did not interfere with the sliding angle measurement.

### Leaching of SNAP from NORel-SR and LINORel -SR tubing

Total leaching of SNAP during the oil swelling and first 24 hours of use were examined at physiological conditions. Nitric oxide releasing tubing were fabricated as described in the Preparation of SNAP Impregnated Tubing section. Phosphate buffered saline (PBS) with 100 mM EDTA was adjusted to a pH of 7.4 was used, where EDTA was used to ensure any metal ions in the PBS solution are neutralized as metallic ions can act as a catalyst for the decomposition of SNAP to release NO. The NO releasing tubing was submerged in 4 mL of PBS-EDTA, and allowed to incubate at 37 °C in a Thermo Fisher water jacketed incubator and was protected from light. At each time point, the concentration of SNAP in the PBS-EDTA buffer was measured using a Thermo Scientific Genysis 10 S UV-Vis Spectrophotometer and reintroduced to the sample container as to not alter the total incubation volume throughout the measurement period. The SNAP molecule has maxima at 340 and 590 nm, corresponding to the S-NO bond^18,47,63^. Absorbance was recorded for each sample, and concentration was determined using a predetermined calibration curve for known concentrations of SNAP in the PBS/EDTA solution. The pure PBS/EDTA solution was used as a blank for all measurements.

Leaching of SNAP from the silicone tubing was repeated during the oil swelling process as well, to ensure large amounts of SNAP were not lost during the 3-day swelling period. Sections of SNAP impregnated tubing were massed and placed in 4 mL of silicone oil. The absorbance spectra of silicone oil were taken to ensure no interference would be seen between the SNAP maxima and the oil. Pure silicone oil was found to have 0.0 absorbance when PBS-EDTA was used as a blank at 340 nm. Therefore, the same calibration curve of SNAP in PBS-EDTA was used for determining SNAP concentration in the silicone oil. Solubility of SNAP in silicone oil was determined by by adding 10 mg/mL and put on a vortex mixer for 2 min. The suspension was then centrifuged (10 min, 3000 rpm), and a 1 mL sample of the silicone oil was taken for UV vis spectroscopy.

### Adsorption of fibrinogen *in vitro*

Levels of protein adhesion were quantified for the fabricated materials using a modified version of a previously reported method^48^. FITC labeled human fibrinogen (13 mg/mL, Molecular Innovations) was diluted to achieve 2 mg mL^−1^ in phosphate buffer solution (pH 7.4). Sections of the various SR tubing were incubated at 37 °C for 30 minutes in a 96 well plate, followed by the addition of the stock protein solution to achieve a concentration of 2 mg mL^−1^ ^48^. During the addition of the stock solution, the tip of the pipette was held below the air-water interface to avoid denaturing of the protein. Following 2 hours of incubation, infinite dilution of the wells’ contents was carried out to wash away the bulk and any loosely bound protein from the materials. Samples were then imaged under an EVOS FL fluorescent microscope to qualitatively assess the degree of protein adhesion on the surface. All images were taken at an equal light intensity.

### Assessment of platelet adhesion *in vitro*

All protocols pertaining to the use of whole blood and platelets were approved by the Institutional Animal Care and Use Committee. Freshly drawn porcine blood was drawn into a BD 60 mL syringe with 3.4% sodium citrate at a ratio of 9:1 (blood: citrate) through a blind draw.

Immediately following the draw, the anticoagulated blood was centrifuged at 1100 rpm for 12 min using the Eppendorf Centrifuge 5702. The platelet rich plasma (PRP) portion was collected carefully with a pipet as to not disturb the buffy coat. The remaining samples were then spun again at 4000 rpm for 20 min to achieve platelet poor plasma (PPP). Total platelet count in both the PRP and PPP fractions were determined using a hemocytometer (Fisher). The PRP and PPP were combined in a ratio to give a final platelet concentration ca. 2 × 10^8^ platelets mL^−1^. Calcium chloride (CaCl_2_) was added to the final platelet solution to achieve a final concentration of 2.5 mM^48^.

Sections of each respective tubing were cut into small sections (0.5 cm long) and placed in a 48 well plate. Approximately 1.5 mL of the calcified PRP was added to each well containing a catheter sample, with one sample per well, and incubated at 37 °C for 90 min with mild rocking (25 rpm) on a Medicus Health blood tube rocker. Following the incubation, the wells were infinitely diluted with 0.9% saline.

The degree of platelet adhesion was determined using the lactate dehydrogenase (LDH) released when the adherent platelets were lysed with a Triton-PBS buffer (2% v/v Triton-X-100 in PBS) using a Roche Cytotoxicity Detection Kit (LDH). A calibration curve was constructed using known dilutions of the final PRP, and the platelet adhesion on the various tubing samples was determined from the calibration curve.

### *In vitro* bacterial adhesion and growth in a continuous flow CDC bioreactor

The ability of the LINORel-SR tubing to prevent bacterial binding and growth on the polymeric surface was tested *in vitro* in a continuous flow CDC bioreactor against gram-positive (*Staphylococcus aureus*) and gram negative (*Pseudomonas aeruginosa*). The use of CDC bioreactor provides a highly favorable environment for bacterial growth and biofilm formation through a continuous supply of nutrients so that antimicrobial efficacy of LINORel-SR tubing can be tested for a prolonged time interval. In this study, we examined the long-term performance of the SNAP-Si oil and control (without SNAP and/or Si-oil coat) tubing in a 7-day model. A single isolated colony of the bacterial strains was incubated overnight in LB medium for 14 h at 150 rpm at 37 °C. The optical density (O.D) was measured at 600 nm (OD600) using UV-vis spectrophotometer. All samples (SR, LI-SR, NORel-SR, and LINORel-SR; N = 3 each) were sterilized with UV irradiation under a Biosafety Cabinet (BSC) and fitted inside the CDC bioreactor. The CDC bioreactor was sterilized using high pressure saturated steam for 30 min at 121 °C in an autoclave. The CDC bioreactor (working volume 1000 mL) with 400 mL of LB medium (2 g L^−1^) was inoculated with the bacterial culture in a manner that the final OD600 falls in the range of 10^7^–10^9^ CFU mL^−1^ to simulate the chronic infection conditions. The CDC bioreactor on one end was connected to a feed bottle having a continuous supply of sterile LB medium (2 g L^−1^) and to a sealed container to collect the wash out in a sterile manner on the other end. After 7 days, samples were removed under a BSC and gently rinsed with PBS, pH 7.4 in order to remove any loosely bound bacteria. The rinsed films were then transferred to a 15 mL tube with 2 mL sterile PBS and homogenized for 60 sec using an OmniTip homogenizer^23^. The shear force from the homogenizer tip ensured the transfer of the bound bacterial strains from the tubing to the PBS solution. Thereafter, serial dilution (10^−1^ to 10^−5^) were made suing sterile PBS and bacterial strains were plated on Petri-dishes solid LB-agar medium using an L-spreader. The antimicrobial efficacy of the LINOrel-SR tubing was measured relative to the SR control rubing using equation 2.

2 $$ \% \,{Bacterial}\,{inhibition}=\frac{(\frac{CFU}{c{m}^{2}}\,in\,control-\frac{CFU}{c{m}^{2}}\,in\,test)\times 100}{\frac{CFU}{c{m}^{2}}\,in\,control}$$

### *In vitro* cytocompatibility

The ability of the NORLI-SR tubing to generate any cytotoxic responses was tested on mouse fibroblast cells (ATCC-1658) using cell counting kit-8 (CCK-8) assay in accordance with ISO 10993 standard. The CCK-8 assay is based on the reduction of highly water-soluble tetrazolium salt. WST-8 [2-(2-methoxy-4-nitrophenyl)-3-(4-nitrophenyl)-5-(2,4-disulfophenyl)-2H-tetrazolium monosodium salt] by dehydrogenases present in viable mammalian cells to give formazan (an orange color product) in direct proportion to the number of viable cells when detected at a wavelength 450 nm. Mouse fibroblast cells were cultured in a humidified atmosphere with 5% CO_2_ at 37 °C in 75 cm^2^ T-flask containing premade DMEM medium (Thermo Fischer) with 10% fetal bovine serum (FBS) and 1% penicillin-streptomycin. After the confluency reached 80–90%, cells were removed from the flask using 0.18% trypsin and 5 mM EDTA, counted using bromphenol blue in a hemocytometer and 100 µL of 5000 cells mL^−1^ were seeded in 96 well plates. The leachates from the each sample (control SR, NORel-SR, LI-SR and LINORel-SR) was obtained by soaking 10 mg of tubing sample in 10 mL DMEM medium for 24 h at 37 °C in the amber vial (N=5 each). To each of the wells containing fibroblast cells, 10 µL of the CCK-8 solution was added and cells with CCK-8 dye were incubated for 3 h. Negative controls containing 5000 cells/ml were grown in 5 separate wells for reference to compare with the cells treated with leachates. Absorbance values were measured at 450 nm and the relative cell viability of mammalian cells exposed to the respective leachates were compared. 100 µL of the DMEM medium without cells was added in 5 of the wells and used as blank to adjust the background interference from DMEM media. Results were reported as percentage cell viability difference between the leachate treated cells relative to the negative control (without leachate treatment) using equation 3.

3 $$ \% \,{Cell}\,{Viability}=\frac{{Absorbance}\,{of}\,{the}\,{test}\,{samples}\,}{{Absorbance}\,{of}\,{the}\,{control}\,{samples}}\times 100$$

### Statistical analysis

Data is reported as the mean ± standard deviation. Statistical significance was determined using a two-tailed t-test assuming unequal variances with α = 0.05. All measurements were conducted with N = 3 samples unless otherwise stated.

### Data availability

The datasets generated during and/or analyzed during the current study are available from the corresponding author on reasonable request.

## Electronic supplementary material

Supplementary Information
