# Supplementary material for: Liquid-infused nitric oxide-releasing (LINORel) silicone for decreased fouling, thrombosis, and infection of medical devices
Source: Sci Rep. 2017 Oct 19;7:13623. doi: 10.1038/s41598-017-14012-9 (PMC5648791; doi:10.1038/s41598-017-14012-9)
Supplement: Supplementary file 1 — Supplementary Information [file 41598_2017_14012_MOESM1_ESM.pdf]

Liquid-infused nitric oxide-releasing (LINORel) silicone for decreased fouling, thrombosis, and infection of medical devices

Marcus J. Goudie[a], Jitendra Pant[a], Hitesh Handa[*a]

[a]School of Chemical, Materials and Biomedical Engineering, College of Engineering, University of Georgia, Athens, GA, USA

Supplementary Information

*Corresponding Author:

Hitesh Handa
University of Georgia
220 Riverbend Road
Athens, GA 30602
Telephone: (706) 542-8109
E-mail: hhanda@uga.edu

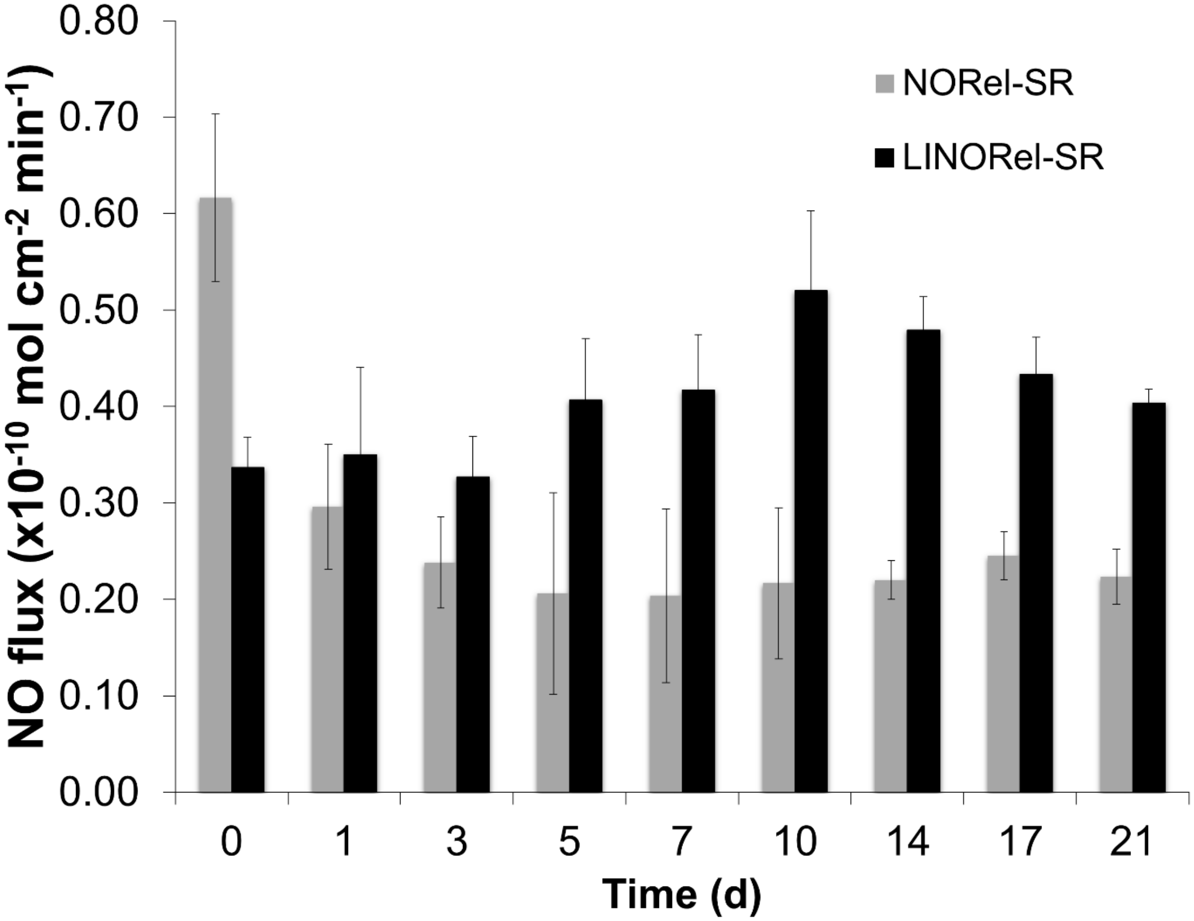

Figure S1: Extended nitric oxide release from LI-SR and LINORel-SR measured via chemiluminescence over a 21 d period.
